# Origin of the Degree of D.D.S.

**Published:** 1903-02

**Authors:** William H. Trueman


### Domestic Correspondence.

### ORIGIN OF THE DEGREE OF D.D.S.

To the Editor:

Sir,—A little while ago you wrote me regarding the origin of the degree Doctor of Dental Surgery. Recently, in connection with another matter, I have had occasion to look it up, and find that it came into being with the American Association of Dental

Surgeons, as part of their plan for the education and elevation of the profession.

The original members were carefully selected by the few who took upon themselves the organization of the society, and sent invitations to a number of dentists in whom they had confidence and who were in their respective communities acknowledged to be reputable and well-qualified practitioners. These came together at New York, on the 18th of August, 1840, at the American Hotel.

They met in convention and organized the Association. (See *American Journal of Dental Surgery,* vol. i., p. 157.)

For the information of the public, in order that they might know who were and who were not recognized by their professional brethren as reputable and well-qualified practitioners, provision was made for giving to those admitted to membership a diploma conferring the degree Doctor of Dental Surgery. All the original members were entitled to this on payment of a fee, and provision was made in Article I. of the By-laws, Section 10, for an Examining Committee who were to meet at least once a year for the examination of applicants for the degree of Doctor of Dental Surgery.

See, also, Article XII., Section 2, of the Constitution (ibid., p. 161, bottom of page), where graduates of a regularly chartered dental college of the United States are exempted from this examination; also, Article I., Section 1, of By-Laws, and Section 5 of the same; Article IV. of the By-Laws; and, finally, page 169, "Eleazar Parmly is by resolution appointed agent of the society to present a petition to the Legislature of the State of New York asking for a charter, with power of conferring the degree Doctor of Dental Surgery."

All this preceded the first dental college.

I am unable to find any record of the society receiving a charter, nor yet of any action of the State Legislature giving to it the power of conferring degrees, and as at a later period the Baltimore college conferred the degree upon some members of the society who were entitled to it by these provisions, it may have been that the society found itself unable to carry out these designs. However that may be, it is beyond question that the degree originated with the promoters of the first National Dental Association.

This may be new to you, and perhaps by lapse of time may now be without interest.

William H. Trueman.

[The foregoing was the outcome of a query sent the writer: "How did the degree D.D.S. originate?" Inquiries of the Baltimore College of Dental Surgery failed to secure a satisfactory answer. It was then referred to Dr. Wm. H. Trueman, with the above result.—Ed.]

